# Relationship Between the Structure of the Flavone *C*-Glycosides of Linseed (*Linum usitatissimum* L.) and Their Antioxidant Activity

**DOI:** 10.3390/molecules29245829

**Published:** 2024-12-10

**Authors:** Imen Ghozzi, Jean-Xavier Fontaine, Roland Molinié, Redouan Elboutachfaiti, Lylia Akkouche, Khaled Sebei, David Mathiron, Christophe Hano, Laurine Garros, Elodie Choque, Romain Roulard, Laurent Petit, Cédric Delattre, Emmanuel Petit, Anthony Quéro

**Affiliations:** 1 UMRT INRAE 1158 BioEcoAgro, BIOlogie des Plantes et Innovation (BIOPI), Université de Picardie Jules Verne, IUT GB, Avenue des Facultés, Le Bailly, 80025 Amiens, France; imen.ghozzi@etud.u-picardie.fr (I.G.); jean-xavier.fontaine@u-picardie.fr (J.-X.F.); roland.molinie@u-picardie.fr (R.M.); redouan.elboutachfaiti@u-picardie.fr (R.E.); lylia.akkouche28@gmail.com (L.A.); elodie.choque@u-picardie.fr (E.C.); romain.roulard@u-picardie.fr (R.R.); laurent.petit@u-picardie.fr (L.P.); anthony.quero@u-picardie.fr (A.Q.); 2 LR22ES04 Bioresources Environment Biotechnology, Institut Supérieur des Sciences Biologiques Appliquées de Tunis, Faculté des Sciences de Tunis, Université Tunis-El Manar, Tunis 2092, Tunisia; khaled.sebei@issbat.utm.tn; 3 Plate-Forme Analytique, Université de Picardie Jules Verne, 33 rue Saint Leu, 80039 Amiens, France; david.mathiron@u-picardie.fr; 4 Institut de Chimie Organique et Analytique, Université d’Orléans-CNRS, UMR 7311, BP 6759, CEDEX 2, 45067 Orléans, France; hano@univ-orleans.fr (C.H.); laurine.garros@univ-orleans.fr (L.G.); 5 Clermont Auvergne INP, CNRS, Institut Pascal, Université Clermont Auvergne, 63000 Clermont-Ferrand, France; 6 Institut Universitaire de France (IUF), 1 Rue Descartes, 75005 Paris, France

**Keywords:** flavone *C-*glycosides, preparative HPLC, NMR, LC-MS, antioxidant activity, structure–activity relationship

## Abstract

Flavonoids have been documented to have good antioxidant activities in vitro. In recent years, reports on the antioxidant activities of flavone *C-*glycosides, a subclass of flavonoids, have attracted great attention. Despite the wealth of information on this subject, the correlation between structure and function is not well understood. In this work, the relationship between the structure and the antioxidant activity of 12 flavone *C-*glycosides extracted from the aerial part of winter linseed (*Linum usitatissimum* L.) was studied to fill the current gaps. Orientin, isoorientin, vitexin, isovitexin, swertisin, swertiajaponin, carlinoside, schaftoside, lucenin-1, lucenin-2, vicenin-1, and vicenin-2 were purified by preparative HPLC and by the drowning-out crystallization method. Then, the control of the purity and the confirmation of the chemical structures were assessed by LC-MS and NMR analyses. The antioxidant activity was evaluated using ABTS, CUPRAC, FRAP, and iron chelating activity in vitro assays. Luteolin and its flavone *C-*glycoside derivatives exhibited higher antioxidant activity than apigenin and its flavone *C-*glycosides derivatives. This could be attributed to the ortho-dihydroxyl groups at C-3′ and C-4′ of the B ring in the flavonoid skeleton, which seemed to play an important role in antioxidant behavior. These results indicate that the antioxidant activity of these compounds, derived from apigenin and luteolin, can be closely related to their structural characteristics, including the position and nature of the sugars, the number of hydroxyl groups, and the presence of methyl group.

## 1. Introduction

Reactive oxygen species (ROS), including the superoxide anion (O_2_^•−^), hydrogen peroxide (H_2_O_2_), and hydroxyl radical (OH^•^), are natural by-products of oxidative metabolism and cellular responses to xenobiotics, cytokines, and bacterial invasion.

Oxidative stress arises from an imbalance between ROS production and the cell’s antioxidant capacity, leading to irreversible damage to biomolecules such as lipids, proteins, nucleic acids, and carbohydrates. This imbalance contributes to cell death and the potential progression of various disease states, including diabetes, cancer, neurodegeneration, aging, and atherosclerosis [1].

Nowadays, natural antioxidants are attracting increasing attention due to the putative cytotoxicity and carcinogenicity of some synthetic antioxidants [2]. Flavonoids, recognized as powerful antioxidants, represent a valuable source of pharmacologically active compounds for drug discovery. Glycosylated flavonoids, in particular, have demonstrated significant beneficial biological effects on human health. Their ability to reduce oxidative stress underlies numerous health benefits [3]. For example, in the field of dermatology, flavonoids exhibit significant photoprotective properties due to their potent antioxidant activity, which mitigates UV-induced oxidative stress in cutaneous tissues. For instance, isoorientin, a *C*-glycosyl flavone, has demonstrated remarkable in vitro antioxidant efficacy. At a concentration of 87 μM, isoorientin significantly inhibited superoxide radical formation and suppressed skin tumor polymerization through xanthine oxidase modulation [4]. Furthermore, swertiajaponin, another *C*-glycosylated flavonoid, has shown robust antioxidant capacity and notable melanogenesis inhibition in human epidermal melanocyte models [5].

Flavonoids are a broad class of low-molecular-weight molecules, characterized by phenolic groups, which act as hydrogen donors to free radicals, stabilizing surplus electrons. The antioxidant activity of flavonoids, which is governed by their chemical structure, is also described for its capacity to transfer electrons to free radicals, activate antioxidant enzymes, chelate metal catalysts, reduce alpha-tocopherol radicals, and/or inhibit oxidases [6]. Other substances, such as flavonols, exert an indirect effect by chelating metal ions that could disrupt cellular redox equilibrium [7].

**Figure 1:**
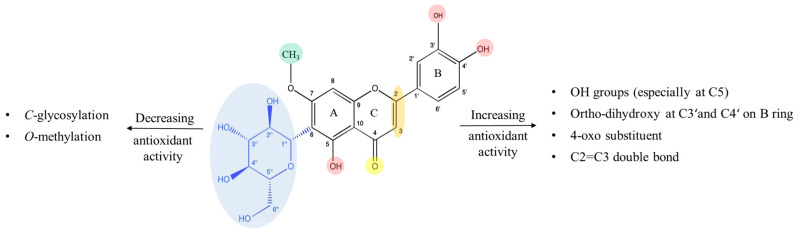
Relationship between flavone *C*-glycoside (swertiajaponin) structure and its antioxidant activity.

The efficacy and bioactivity of these compounds are significantly influenced by their molecular structure, particularly the number and positions of hydroxyl groups, as well as the nature and extent of various structural modifications as well as glycosylation and methylation. Previous studies have suggested that the existence of hydroxyl groups, particularly in the ring B of the flavonoid structure, along with the presence of a C2=C3 double bond in conjunction with a C4-carbonyl group, enhances antioxidant properties (Figure 1). However, the presence of saccharide groups tends to diminish its antioxidant activity [8]. Despite glycosylation typically reducing the capacity of flavonoids to inhibit free radicals compared to their aglycone (luteolin or apigenin), flavone *C*-glycosides have captured the attention of researchers. This subclass of flavonoids has been the focus of numerous scientific studies in recent years.

The authors have shown that the isoorientin (luteolin 6-*C-*glucoside) and the orientin (luteolin 8-*C*-glucoside) exhibited strong antiperoxidative activity (in which free radicals attack lipids in cell membranes, leading to cell damage) with an EC50 value of 9.5 µM in the DPPH assay [9]. Treatment with vicenin-2, a flavone *C*-diglycoside, significantly restored antioxidant enzymatic activitiessuch as superoxide dismutase (SOD), catalase, glutathione peroxidase (Gpx), and glutathione (GSH), thereby protecting cells from UVB-induced oxidative stress, demonstrating the beneficial effect of vicenin-2 in neutralizing and scavenginghydroxyl or DPPH radicals [10]. Isoorientin demonstrated the highest antioxidant activity to DPPH assay, along with potent inhibition of xanthine oxidase and 15-lipoxygenase, with EC50 values of 18.1, 117.2, and 86.4 µM, respectively [11]. The characteristic that draws the attention of researchers to flavone *C*-glycosides is a carbon–carbon bond between the glycosyl group and the aglycone part, making them more stable during enzymatic or acidic hydrolysis [12]. However, there is little information available on the bioactivity of flavone *C-*glycosides and the relationship between their structure and function. Furthermore, various mechanisms of flavone *C*-glycosides’ action and the different methods used to measure in vitro oxidative processes contribute to incoherent and incomparable data.

*Linum usitatissimum* L., an ancient plant with well-established medicinal properties, continues to be cultivated today. It is primarily grown for its stems, which are a source of fiber (fiber flax) used in textile production, and for its seeds (linseed), which contain an edible oil rich in n − 3 fatty acids, primarily *α*-linolenic acid. Today, there is increasing interest in other parts of the linseed plant and this traditional dual-purpose crop has the potential to develop into a multi-purpose crop [13]. Although the phenolic compounds found in other parts of linseed have been less studied, our understanding of them remains limited. However, as early as 1971, Dubois and Marby identified mono- and di-*C*-glycosyl flavones, derivatives of apigenin and luteolin, in flax leaves and stems using nuclear magnetic resonance (NMR) [14]. Since, new *C-*glycosyl flavones have been identified in leaves from winter flax varieties and spring flax varieties by conducting additional analysis using NMR and liquid chromatography-mass spectrometry (LC-MS) techniques [15].

In this context, the objective of our study was to investigate the antioxidant activity and to elucidate the structure–activity relationships of flavone *C*-glycosides, major phenolic compounds identified in extracts of aerial parts of linseed. To achieve this, flavone *C*-glycosides were isolated from *Linum usitatissimum* L. and identified through NMR and LC-MS. The antioxidant activities of the isolated compounds were assessed using in vitro methods—ABTS, CUPRAC, FRAP, and iron chelating activity—to evaluate diverse antioxidant mechanisms.

## 2. Results

### 2.1. Obtaining and Characterization of Crude Extract

The extraction of plant material for experimental purposes is an important step and a key factor in achieving quality research results. This involves determining the quantity and quality of bioactive compounds before proceeding with the planned biological tests. In this study, the maceration of 500 g of aerial part of *L. usitatissimum* in methanol–water (1:1) yielded 103.4 ± 7.4 g of crude extract, approximately 20% of the initial plant powder mass. The characterization of the crude extract provided valuable insights into its composition. Notably, the water content at 17 ± 1.3% indicated moisture, while the ash content at 16 ± 3.5% revealed the presence of inorganic residues. A content of 6% in protein was determined by the Kjeldahl method.

**Figure 2:**
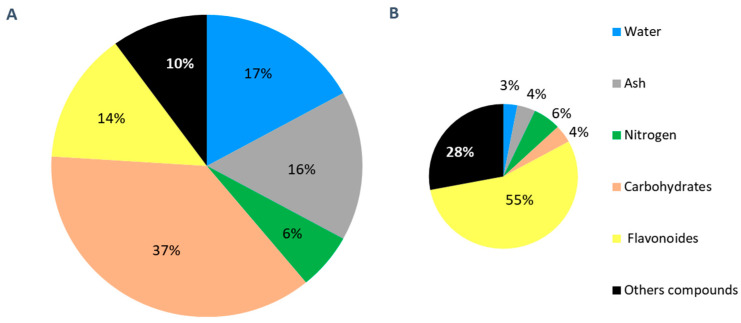
The category and percentage of main components in the crude extract (**A**) and in the fraction enriched in flavone *C-*glycosides (**B**).

The proportion of carbohydrates within the crude extract was 37%. Regarding the composition of carbohydrates, HPAEC-PAD analysis identified sucrose as the predominant carbohydrate, comprising 17% of the total. Additionally, glucose was present at 14%, myo-inositol at 4%, and fructose was the least abundant monosaccharide, constituting only 2% (Supplementary Materials S1). Finally, the quantification of total flavonoids, as described earlier, revealed a content of 14 ± 0.7% (Figure 2A).

### 2.2. Obtaining and Characterization of a Fraction Enriched in Flavone C-Glycosides

Compared to the crude extract, the fraction enriched in flavone *C*-glycosides yielded only 20.8 ± 0.8 g, approximately 4% of the initial plant powder mass. As shown in Figure 2B, after purification using XAD-16N resin, the ash content significantly decreased from 16 ± 3.5% to 4 ± 3.5%, and the water content also showed a notable reduction from 17 ± 1.3% to 3 ± 0.6%. However, the protein content was the same for both the crude extract and the fraction enriched in flavone *C*-glycosides, each containing 6%. However, the obtained mass of the fraction enriched in flavone *C*-glycosides being five times lower than that of the crude extract implies that the purification step led to the removal of 80% of nitrogen compounds from the crude extract. However, we assume an overestimation of the protein content in XAD extract. Indeed, the Kjeldahl method does not provide a precise identification of the nature of nitrogen compounds, due to the necessity, for example, to carry out two analyses to determine the difference between non-protein nitrogen and total protein nitrogen. Furthermore, in our study we used a conversion factor of 6.25 between nitrogen and protein content. Thus, considering that plants are composed of amino acids and the XAD resin selectively retains aromatic compounds, we could consider that the nitrogen compounds present in the XAD extract correspond to aromatic amino acids (tryptophan, phenylalanine, and tyrosine). In contrast, the 6% nitrogen compounds in the crude extract are assumed to be represented by a broader range, including amino acids, proteins and phenolic compounds with nitrogenous bases.

For carbohydrates, the content in the fraction enriched in flavone *C-*glycosides decreased significantly from 37% to 4%. More specifically, the content of sucrose decreases from 17% to 2%, the content of glucose from 14% to 1%, the content of myo-inositol from 4% to 1%, and the content of fructose from 2% to 0.2% (Supplementary Materials S1). By contrast, the ratio of flavonoids significantly increased from 14 ± 0.7% in the crude extract to 55 ± 2% in the fraction enriched in flavone *C-*glycosides. This result suggests that the XAD resin retains all the flavonoids. To confirm this result, HPLC analyses were conducted on the liquid extract both before and after passage through the XAD resin. The volume and thus the concentration of both extracts were the same; this allowed for a direct comparison of the areas obtained from the HPLC analysis.

**Figure 3:**
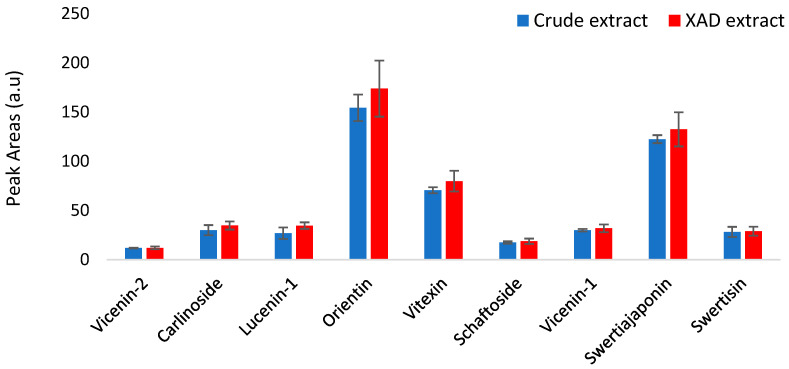
Qualitative analysis of the composition (flavone *C-*glycosides) of the aerial part of linseed extract before and after treatment with XAD-16N resin (*n* = 3).

The main UV signals obtained in both fractions were attributed to nine flavone *C*-glycosides. Based on the peak area (%) measured for each compound, the content of all nine compounds was similar in both fractions, as seen in Figure 3. For example, the contents of swertisin and vicenin-2 were 102% in the extract obtained after pre-purification using the XAD resin versus to crude extract. The results confirm the effectiveness of purification using the XAD-16N resin, enabling the retention of all target compounds while eliminating 80% of the initial mass. This step serves to concentrate the flavone *C*-glycosides by removing non-desirable components, including nitrogen compounds, water, sugars, and mineral elements. Removing these components could concentrate flavone *C*-glycosides in the extract and simplify the subsequent purification process.

### 2.3. Optimization of Liquid–Liquid Extraction to Simplify the Matrix Before Purification

Appropriate two-phase solvent system plays an important role in successful separation of the target compounds. A solvent system composed of H_2_O/EtOAc/n-BuOH was chosen to carry out the liquid–liquid extraction and optimization trials were conducted at different ratios.

**Figure 4:**
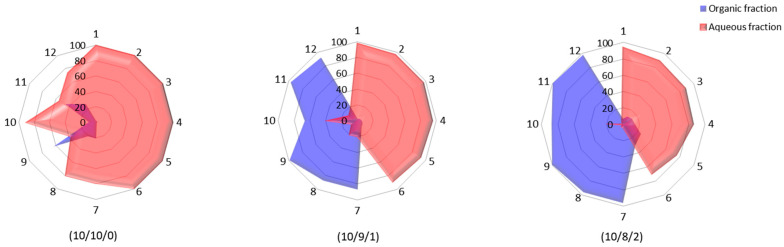
Extraction yields obtained using different ratios of H_2_O/EtOAc/n-BuOH. (1) Lucenin-2; (2) vicenin-2; (3) carlinoside; (4) lucenin-1; (5) schaftoside; (6) vicenin-1; (7) orientin; (8) isoorientin; (9) vitexin; (10) swertiajaponin; (11) isovitexin; and (12) swertisin (*n* = 3 for 10/10/0 and 10/8/2 and *n* = 2 for 10/9/1).

As shown in Figure 4, the LC-MS analysis revealed that during separation more than 98% of all flavone *C*-diglycosides were isolated in the aqueous phase using the solvent system H_2_O/EtOAc/n-BuOH at a ratio of 10/10/0. In contrast, for flavone *C-*monoglycosides, 73.3 ± 4.8% of swertisin, 78.7 ± 3.3% of orientin and isoorientin, and 90.5 ± 1.7% of swertiajaponin were accumulated in the aqueous phase. Only vitexin (62.6 ± 10.2%) and isovitexin (45.4 ± 7%) were detected in significant quantities in the organic phase. These results indicate that the H_2_O/AcOEt solvent system is not effective for the separation of target compounds. Consequently, n-BuOH was incorporated into the extraction solvents using H_2_O/EtOAc/n-BuOH at a ratio of 10/9/1. This adjustment resulted in improved solubility and affinity of flavone *C*-monoglycosides for the organic phase, as demonstrated by the increased extraction yields compared to the initial solvent system tested: 86.5 ± 5.9% for orientin and isoorientin, 98.8 ± 0.6% and 97.3 ± 1.8% for vitexin and isovitexin, respectively, and 91.7 ± 4.3% for swertisin. This time, only 38.5 ± 1.7% of swertiajaponin was accumulated in the aqueous phase. On the other hand, the presence of n-BuOH led to a slight reduction in the extraction yield of flavone *C*-diglycosides, with percentages ranging between 89.2 ± 3% and 97.5 ± 0.7% in the aqueous fraction. A third extraction system was therefore tested, focusing on a higher proportion of n-BuOH (H_2_O/EtOAc/n-BuOH at 10/8/2). This solvent system effectively separated flavone *C*-diglycosides and flavone *C-*monoglycosides, with the latter showing improved affinity for the organic phase. The third extraction system demonstrated a significant improvement in the extraction yield of flavone *C*-monoglycosides in the organic phase compared to the first two solvent systems. The results obtained were as follows: 87 ± 1.4% for swertiajaponin, 97.6 ± 0.3% for swertisin, 96.1 ± 0.4% for orientin and isoorientin, 99.2 ± 0.1% for vitexin, and 99.1 ± 0.3% for isovitexin. These high extraction yields, all exceeding 80%, indicate the superior efficiency of the third system in isolating flavone C-monoglycosides. For flavone *C*-diglycosides, there was a loss of between 6.1 ± 1.4% and 30 ± 2.8% in the organic phase. However, their extraction yield remained satisfactory, ranging from 70 ± 2.8% to 93.9 ± 1.4% in the aqueous phase. This third extraction system (H_2_O/EtOAc/n-BuOH at 10/8/2) was ultimately chosen, prioritizing the purification of flavone *C*-monoglycosides, despite some loss of flavone *C*-diglycoside compounds.

### 2.4. Evaluation of the Purity and Identification

The organic phase and the aqueous phase fraction compounds were successfully separated and purified using preparative HPLC and the drowning-out crystallization method, as explained in Section 4.5 and Section 4.6. It was a great challenge to isolate the flavone *C*-glycosides and their isomers simultaneously in the present study. As shown in Table 1, the LC-MS data revealed purity levels ranging from 93.3 ± 0.1% to 99.8 ± 0.2%, while the NMR data revealed purities greater than 95% for all compounds.

**Figure 5:**
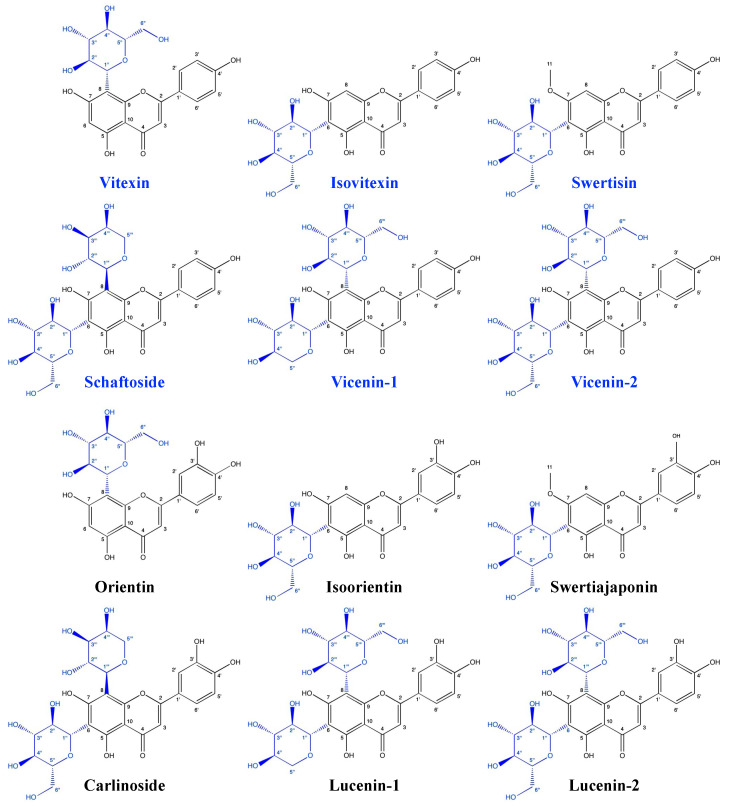
Structures of the flavone *C*-glycosides derived from luteolin (name in black) and apigenin (name in blue) isolated from the aerial part of *L. usitatissimum*. L.

The identification of compounds **1**–**12** was performed using LC-MS analysis (Supplementary Materials S2) and ^1^H NMR, ^13^C NMR analysis (Supplementary Materials S3). These data were compared with published data [16,17,18,19,20,21,22,23] and the structures are shown in Figure 5.

### 2.5. Antioxidant Activity

**Figure 6:**
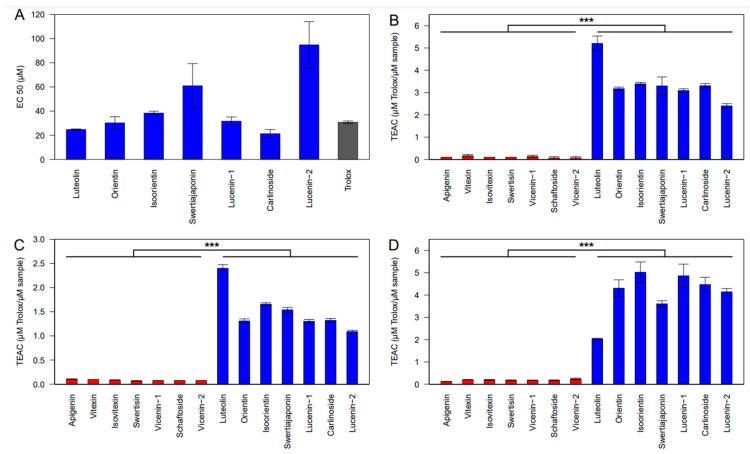
The antioxidant capacity of flavone *C*-glycosides extracted from *Linum usitatissimum* L. (**A**) ABTS; (**B**) CUPRAC; (**C**) FRAP; and (**D**) iron chelation. EC50 (µM)/TEAC (µM Trolox/µM sample) values were calculated using different concentrations expressed as means ± SD (*n* = 3, *** *p* < 0.001).

Antioxidants can neutralize free radicals primarily through two mechanisms: hydrogen atom transfer (HAT) and single electron transfer (SET). However, it is rare for reactions to follow exclusively one mechanism or the other. In this study, the antioxidant capacity of the obtained compounds was determined using four different methods: the ABTS radical cation (ABTS^•+^), the CUPRAC, the FRAP, and the ferrous-iron chelating assay. As shown in Figure 6A and in Supplementary Materials S4, carlinoside exhibited the strongest ABTS^•+^ scavenging activity with an EC50 value of 21.2 ± 3.5 µM, followed by luteolin with an EC50 value of 24.8 ± 3.5 µM and orientin with an EC50 value of 30.2 ± 5.1 µM. In comparison, the positive control, Trolox, had an EC50 value of 30.7 ± 1.1 µM, followed by lucenin-1 with an EC50 value of 31.7 ± 3.5 µM and isoorientin with an EC50 value of 38.3 ± 1.6 µM. Although swertiajaponin and lucenin-2 showed weaker ABTS^•+^ scavenging activity, there was a remarkable increase in their EC50 values, which were 61 ± 18.4 µM and 94.8 ± 19.3 µM, respectively. The radical scavenging activity against ABTS of luteolin and its derivatives was found to be much higher than that of apigenin and its derivatives, which showed little to no activity. The antioxidant compounds shown in Figure 6B demonstrate that the highest capacities.

In the CUPRAC method were observed in the following order luteolin > isoorientin > carlinoside > swertiajaponin > orientin > lucenin-1 > lucenin-2. In this series, luteolin exhibited the highest TEAC_CUPRAC_ value of 5.2 ± 0.06 µM Trolox/µM, indicating its potent antioxidant activity. Isoorientin, carlinoside, swertiajaponin, orientin, and lucenin-1 showed close TEAC_CUPRAC_ values, ranging from 3.4 to 3.1 µM Trolox/µM, while apigenin and its *C*-glycosylated derivatives like vitexin and isovitexin did not show significant capacities for reducing copper ions (Cu^+^).

FRAP value was used as an important indicator for the antioxidant activity, and FRAP results of the 14 compounds are presented in Figure 6C. The total TEAC_FRAP_ values varied from 2.4 to 0.07 μM Trolox/µM. Luteolin, isoorientin, swertiajaponin, carlinoside, orientin, lucenin-1, and lucenin-2 were the top seven compounds with highest ferric-reducing antioxidant activity, 2.4 ± 0.05, 1.6 ± 0.02, 1.5 ± 0.03, 1.32 ± 0.02, 1.31 ± 0.03, 1.3 ± 0.02, and 1 ± 0.02 μM Trolox/µM, respectively. Moreover, apigenin and its derivatives had the lowest ferric-reducing capacity. The TEAC_FRAP_ values for the seven compounds had the following order: apigenin > vitexin > isovitexin > vicenin-2 > vicenin-1 > schaftoside > swertisin. Nevertheless, the *C*-glycosyl flavones derived from luteolin showed remarkable antioxidant activity in the iron-chelation assay, with TEAC values ranging from 5 to 3.6 μM Trolox/µM, with isoorientin showing the highest value and swertiajaponin the lowest. These TEAC values were higher than those of their aglycone, luteolin (2 ± 0.02 µM Trolox/µM). Moreover, in the concentration range we tested, apigenin and its derivatives showed very low iron-chelation activity even at the highest concentration compared to luteolin and its derivatives (Figure 6D).

## 3. Discussion

In recent years, the cultivation of *Linum usitatissimum* (flax or linseed) has generated increasing interest. The utilization of this plant is expanding, and the presence of flavone *C-*glycosides has particularly attracted the attention of industrials. Various flavone *C*-glucosides were found in *Linum usitatissimum*, including orientin and isoorientin and their derivatives [24]. For example, flax straw was found to be a rich source of valuable metabolites, including *C*-glycosylated flavones [25]. Since then, the content of these specialized metabolites has been monitored throughout the development of plant. These studies have shown that flavones are mainly accumulated during flowering [26,27]. Additional research has revealed that flavone *C-*glycosides are the predominant phenolic compounds in the aerial part of flax. The antioxidant activity of flax extracts has been evaluated at different stages of development, suggesting that the aerial part of flax at the flowering stage or during seed ripening represents a valuable source of flavone *C*-glycosides for their nutraceutical properties or as functional food compounds [26].

Most research on linseed has traditionally focused on the seed. However, studies on its aerial parts remain relatively limited. In this context, we propose a method to obtain linseed extracts enriched in flavone *C-*glycosides. This method involves the use of XAD-16N resin and is inspired by research conducted on other plant species. In fact, in *Glycyrrhiza glabra,* it has been noted that XAD-16N resin effectively separated flavonoids and significantly enhanced their antioxidant activity. Specifically, the antioxidant activity of the flavonoids after purification by XAD-16N resin was 2 to 3 times higher than that of the extract obtained before passing through the resin, indicating that the XAD resin effectively optimized the antioxidant activity by concentrating the bioactive compounds [28].

In linseed, our studies have demonstrated that XAD-16N concentrates flavone *C*-glycosides by a factor close to 4 compared to the crude extract. The use of this method combined with liquid–liquid extraction has facilitated the purification of the 12 major flavones *C*-glycoside of linseed by preparative HPLC and the drowning-out crystallization. In liquid–liquid extraction, the H_2_O/EtOAc/n-BuOH solvent system at a ratio of 10/8/2 proved to be effective in providing a good partition for the target compounds following the XAD resin step. This finding is consistent with the research conducted by [29], who demonstrated that by combining pre-purification using macroporous resin followed by liquid–liquid extraction using the solvent system EtOAc/n-BuOH/H_2_O at a ratio of 1/2/3 (*v*/*v*/*v*), flavonoid glycosides with high purity were successfully obtained from *Nelumbo nucifera* Gaertn. Additionally, previous studies discussed the preparative isolation of flavone *C*-glycosides from plants. Zhang et al. performed the isolation and purification of four flavone *C*-glycosides (orientin, isoorientin, vitexin, and isovitexin) from bamboo leaves using resin-based column chromatography, preparative HPLC and recrystallization [30].

In any biomedical and chemical context, a truthful description of chemical constitution requires consideration of both structure and purity.

Thus, for these isolated flavone *C*-glycosides compounds, the structure and purity were confirmed through NMR and MS data analysis. It is generally accepted that a purity level greater than 95% is considered reassuring. However, the *Journal of Medicinal Chemistry* requires a purity of >95% for all tested compounds to ensure that the observed effects are accurate and not due to highly active impurities present in the test sample [31].

These 12 flavones, derived from apigenin and luteolin, exhibit diverse structural features that allow understanding the structure–function relationships relating to their antioxidant activity. For example, comparing orientin and isoorientin facilitates the investigation of how sugar position affects antioxidant activity. Similarly, comparing isoorientin and swertiajaponin enables the study of antioxidant activity after the addition of a methyl group to a compound. The comparison between lucenin-1 and lucenin-2 can assess the effect of sugar type (xylose versus glucose at position 6). Lastly, comparisons between vitexin and orientin, isovitexin and isoorientin, swertisin and swertiajaponin, vicenin-2 and lucenin-2, vicenin-1 and lucenin-1, and schaftoside and carlinoside can elucidate the effect of an -OH group on antioxidant activity. This description of combinations provides only a partial view of the possible comparisons. In total, 22 combinations are possible (2 to study the addition of -CH_3_, 2 for sugar position, 4 for sugar type, 6 for the addition of -OH, and 8 for sugar addition). Therefore, linseed serves as a reservoir of interesting natural compounds to provide a better understanding of the relationship between the structure of flavone *C*-glycosides and their antioxidant activity.

Antioxidant assays can be classified in different ways, one of which is based on the reaction mechanism: hydrogen atom transfer (HAT), single electron transfer (SET), or mixed HAT/SET. An antioxidant may operate directly by scavenging stable free radicals (e.g., ABTS), reducing metal ions (e.g., FRAP and CUPRAC), or indirectly by chelating pro-oxidant metals (e.g., iron) or inducing antioxidant enzymes to inhibit oxidative damage [32]. The ABTS assay is based on the scavenging of a blue–green chromophore, the ABTS^•+^ stable radical cation. In this assay, HAT, ET, and proton-coupled electron transfer (PCET) mechanisms may play different roles in varying proportions, depending on the specific reaction conditions such as pH and solvent. In the CUPRAC SET-based assay, the reactive Ar-OH groups of polyphenols and other antioxidants are oxidized to the corresponding quinones, and Cu^2+^–neocuproine is reduced to the Cu^+^–neocuproine complex, which is intensely yellow–orange with a maximum absorption peak at 450 nm. Moreover, the CUPRAC chromogenic redox reaction is carried out at a pH of 7.0, which is close to physiological pH. Therefore, the results obtained from the CUPRAC assay may more accurately reflect the putative in vivo reactions of antioxidants. The FRAP test is a non-radical SET-based method that measures the reduction of the ferric ion Fe^3+^-tri-pyridyltriazine (TPTZ) complex to the intensely blue chelated ferrous Fe^2+^-TPTZ complex by antioxidants in acidic environments (pH = 3.6). The ABTS and CUPRAC tests can measure both hydrophilic and lipophilic antioxidants, the FRAP only measure hydrophilic antioxidants [33].

Fe^2+^ ions produce •OH radicals, which are highly reactive and contribute significantly to oxidative stress through the Fenton reaction. The resulting hydroxyl radicals lead to cellular damage. Chelating agents can bind to metal ions, forming stable complexes and preventing them from participating in reactions that generate free radicals. The iron chelation assay is based on the absorbance measurement of the Fe^2+^-ferrozine complex. The decrease in absorbance of the solution after the introduction of the test sample is related to the metal chelation capacity of the sample [34].

In this study, antioxidant activity of flavone *C*-glycosides under four spectrophotometric assays is reported for the first time. However, investigating antioxidant activity is complex, as no single method can fully depict the natural reactions occurring in vivo and the variations that occur make it difficult to compare the results of different studies.

As shown in our results, luteolin and its derivatives exhibited the highest antioxidant activity, while apigenin and its *C*-glycosides showed little to no activity. This demonstrates that antioxidant activity is influenced by the number of hydroxyl groups on the B-ring, as luteolin and its *C-*glycosylated derivatives feature two hydroxyl groups at positions C-3′ and C-4′ on the B-ring. In contrast, apigenin and its derivatives exhibit only one hydroxyl group at position C-4′ on the B-ring, as shown in Figure 1.

This finding is in agreement with that noted by Zielińska and Zieliński [35], where isoorientin showed a high capacity to scavenge ABTS^•+^ radicals, followed by orientin, while vitexin and isovitexin showed very weak activity. In another study, isoorientin and orientin isolated from the shoot system of *Okinawa taumu* showed significantly higher antioxidant activity against DPPH, with EC50 values of about 31–38 μM, compared to vitexin and isovitexin, which showed EC50 values of 129 and 325 μM, respectively [23]. In 1996, researchers showed that the antioxidant activity of luteolin to scavenge ABTS^•+^ radicals (TEAC = 2.1) was higher than that of apigenin (TEAC = 1.45) [36]. Lucenin-2 isolated from *Stipagrostis plumosa* showed 94% inhibition of the DPPH radical, compared to 13.8% inhibition by vicenin-2 [37].

This clearly indicates that the free hydroxyl groups on the B-ring of the compounds play a key and major role in antioxidant properties. This relation between structure and antioxidant activity of flavonoids was supported by Balasundram et al. [38].

In this study, the presence of a methyl group at the C-7 position of the A ring in swertiajaponin and swertisin influences their antioxidant capacity compared to the other compounds, specifically their ability to scavenge free radicals. Our results are in accordance with a report on the antioxidant activity of some flavone *C*-glycosides extracted from *Cymbopogon citratus*, which indicated that swertiajaponin had a weak ability to quench DPPH, suggesting that the methylated hydroxyl group at C-7 reduced its antioxidant activity [9]. However, the methylation had no effect on the electron transfer mechanism since, in both the CUPRAC and FRAP tests, swertiajaponin showed strong antioxidant activity.

The ABTS, CUPRAC, and FRAP assays indicated that the substitution of glycosyl moieties at the C-6 and/or C-8 positions on the A ring of luteolin decreased the antioxidant activity of *C-*glycosylated flavones. The TEAC_ABTS_ value determined in another study indicated that the antioxidant activity of orientin and isoorientin decreased compared to that of luteolin. Similarly, the scavenging activity of vitexin and isovitexin against ABTS radicals was lower than that of apigenin [35]. A few studies have been conducted on the antioxidant activity of flavone *C-*glycosides using different assays. However, the findings of Apak et al. [39] confirm that the precursor of flavone, naringenin, exhibits significantly higher TEAC_CUPRAC_ (2.28) and TEAC_ABTS_ (1.5) values compared to its corresponding glycoside, naringin, which had TEAC_CUPRAC_ (0.13) and TEAC_ABTS_ (0.2) values.

In the literature, most of the antioxidant activity has been attributed to *C*-glycosyl flavonoids rather than *O*-glycosides. In the FRAP assay performed on the isolated fraction, the fraction concentrated in *C*-glycosylated flavones contributed the most, being responsible for up to 50% of the activity, followed by *O-*glycosyl flavanones (33%) [40].

Antioxidant activity often decreases with the presence of a sugar substituent in the structure, which causes steric hindrance. This effect can be explained by the torsion angle and loss of coplanarity of the aglycone (luteolin or apigenin) and the ability to delocalize electrons. Despite this finding, the authors mentioned that the glycosyl fragment lends hydrophilicity. Moreover, the glycosylation and methylation of hydroxyl groups attenuate the prooxidant activity of flavonoids [6] and provide their stability [41].

On the other hand, derivatives of luteolin and those derived from apigenin have a stronger iron chelation capacity than their respective aglycones. This test evaluates the ability of a compound to bind and chelate metal ions such as iron, forming stable complexes. This chelation can inhibit pro-oxidant reactions involving metals. *C*-glycosyl flavones, due to their bulkier glycosylated structure, can form more stable chelation complexes with iron than aglycones, which explains their superior chelating activity. The Fe^2+^ chelation activity is attributed to the presence of two potential chelating sites. The 5-hydroxy-4-oxo and 3′, 4′-dihydroxyl groups are potential iron chelation sites [42].

It is recognized that structural features determine antioxidant activity. The presence of hydroxyl groups at the 3′ and 4′ positions of the B ring, the double bond between C2 and C3, and conjugation with the 4-oxo group in the C ring have been reported to enhance also the antioxidant activity of flavone *C*-glycosides, while the hydroxyl groups at positions 5 and 7 of the A ring appear to be less critical.

In summary, aglycones appear to be better direct antioxidants as electron donors (CUPRAC, FRAP) or free radical scavengers (ABTS), while glycosylated derivatives seem to be better indirect antioxidants, likely forming more stable chelation complexes with iron. These distinct antioxidant properties can explain the different results obtained for these compounds in these four tests.

In addition to glycosyl substitution, the position and the number of sugars may also affect the antioxidant activity. Orientin and its isomer isoorientin showed close EC50 and TEAC values. In the ABTS test, orientin was more effective, whereas in the CUPRAC, FRAP, and iron chelation tests, isoorientin was more effective. This suggests that the position of the sugar at C6 or C8 has little effect on antioxidant activity but differentiates between the mechanisms of action. On the other hand, carlinoside (luteolin-6-*C*-glucose-8-*C*-arabinoside) and lucenin-1 (luteolin-6-*C*-glucose-8-*C*-xylose) demonstrate significantly higher antioxidant activity than lucenin-2 (luteolin 6,8-di-*C*-glucoside), thereby suggesting that the antioxidant activity of these compounds is mostly dependent on the type of sugar at C-6 and C-8 and the length of the carbon chain of the sugar. In linking sugar chemistry to the biological properties of compounds, we suggest that the sugar’s cyclic form—whether pyranose (a six-membered ring) or furanose (a five-membered ring)—can affect the spatial arrangement and steric hindrance of the glycosidic compounds. Understanding the role of these cyclic structures could provide deeper insights into why certain sugars exhibit different effects compared to others. This conclusion was supported by the established structure–activity relationships for antioxidant compounds [43].

## 4. Materials and Methods

### 4.1. Plant

Winter linseed, *L. usitatissimum* L. var. Angora, was supplied by LINEA, flax breeding company (Grandvilliers, France). The plants were harvested at the beginning of the flowering stage in May 2020. Immediately after harvesting, the plant material was frozen and subsequently lyophilized using a freeze-dryer. The aerial part of *L. usitatissimum* L. was powdered before the extraction process.

### 4.2. Chemical Reagents

Solvents used for extraction (methanol, ethyl acetate, butanol, and acetic acid) were of the analytical purity grade. Standard compounds, luteolin and apigenin, were provided by HWI Pharma Services GMBH (Rülzheim, Rheinland-Pfalz, Germany).

### 4.3. Extraction and Pre-Purification

First, 500 g aerial parts of winter linseed were extracted with MeOH/H_2_O solution (1:1) at room temperature for 1 h and filtrated with a Büchner funnel. The extract was concentrated under reduced pressure. Then, 2 L of the crude extract was adsorbed on an Amberlite XAD-16N resin, washed first with H_2_O to remove unbounded molecules, and then with 80% aqueous EtOH to elute phenolics compounds. The flavonoid fraction eluted with 80% aqueous EtOH and pre-purified by the resin has been named the XAD extract. After lyophilization, 10 g of XAD extract was dissolved in H_2_O and then partitioned successively with ethyl acetate and n-butanol (AcOEt/n-BuOH) three times. The organic phase and the aqueous phase were evaporated under reduced pressure and lyophilized separately.

### 4.4. Characterization of Crude and XAD Extract

#### 4.4.1. Determination of the Ash Content

The ash content of the extracts was determined at 600 °C using a conventional laboratory furnace after drying the samples (2 g). The samples were heated for 40 min at 300 °C and for 150 min at 600 °C. The mass of the ashes resulting from the combustion of organic compounds is expressed as a percentage of the initial mass of the sample:

Ash Content (%) = (Ash Mass/Sample Mass) × 100

#### 4.4.2. Determination of Water Content

First, 1 g of the sample was inserted into the standard containers and measured. After the measurements, the sample was dried in the oven for hours at 105 °C. Once drying was complete, the sample was then weighed. The difference between the two weights represents the mass of water evaporated (M) [44]. M was then calculated as:

M = ((fresh weight − dry weight)/fresh weight) × 100(1)

#### 4.4.3. Determination of Total Nitrogen by the Kjeldahl Method

The protein content was quantified according to the Kjeldahl method. Briefly, samples were weighed and transferred into Kjeldahl digestion flasks (BUCHI SpeedDigester K-425, BUCHI, Tokyo, Japan) containing concentrated H_2_SO_4_ and catalyst (copper sulfate (CuSO_4_)). After digestion, the solution was heated during the distillation process (BUCHI K-350, BUCHI, Tokyo, Japan) to release ammonia gas, which was trapped as ammonium borate in a boric acid solution containing a color indicator. Nitrogen contents were determined by titration with sulfuric acid at 0.02 mol/L until the initial color returned. A conversion factor of 6.25 was used to obtain the protein content.

#### 4.4.4. Dosage of Total Flavonoids

The total flavonoid content was determined using the aluminum chloride colorimetric method as described by Liu [45]. Briefly, 250 mg/L of samples were dissolved in water. This solution (600 µL) was then mixed with 600 µL of 2% aluminum chloride hexahydrate (AlCl_3_). After incubation at room temperature for 60 min, the absorbance of the reaction mixture was read at 420 nm. Results are expressed as rutin equivalent (linear standard range between 5–200 µg/mL). The levels of total flavonoid contents were determined in triplicate.

#### 4.4.5. High-Performance Anion Exchange Chromatography with Pulsed Amperometric Detection (HPAEC-PAD) for Carbohydrate Analysis

The composition of monosaccharides was analyzed using high-performance anion-exchange chromatography with pulsed amperometric detection (HPAEC-PAD) (Dionex Corporation, Sunnyvale, CA, USA). The sample injection volume was 5 μL, with a concentration of 0.2 g/L. The monosaccharides were separated and analyzed utilizing a CarboPac PA–10 column (2 × 250 mm, Dionex) and a CarboPac PA–10 guard column (2 × 50 mm, Dionex). The eluent was pumped at a rate of 0.25 mL/min, and the mobile phase consisted of 50 mM NaOH for the initial 20 min, followed by 200 mM NaOH for the subsequent 10 min, and finally returning to 50 mM NaOH for the last 20 min. A mixture of glucose, fructose, saccharose, and myo-onisitol was used as the reference. Data processing and analysis were performed using Chromeleon 6.8. Chromatographic conditions were used as described by [46].

### 4.5. Preparative HPLC Separation

The aqueous and organic fractions were separated through preparative high-performance liquid chromatography (HPLC). The chromatographic separation was conducted using an LC-8A system (Shimadzu, Marne-la-Vallée, France). The separation was achieved with a Luna^®^ Omega C18 column (Phenomenex, Torrance, CA, USA, 250 mm × 21.2 mm × 5 µm) or a Kinetex^®^ Biphenyl column (Shimadzu, Kyoto, Japan, 250 mm × 21.2 mm × 5 µm).

Generally, 2 mL samples (10 g/L for organic fraction and 15 g/L for aqueous fraction) were injected and eluted with a mobile phase consisting of 0.2% (*v*/*v*) acetic acid in water (A) and methanol (B) at a flow rate of 17 mL/min. The column temperature was set at 30 °C, and detection was performed at a wavelength of 280 nm. The optimized gradient elution conditions were as follows:

For organic fraction: (0–15 min, 35% B; 15–30 min, 35–50% B; 30–33 min, 50–90% B; 33–37 min, 90% B; 37–40 min, 90–35% B; 40–50 min, 35% B).

For aqueous fraction: (0–12 min, 25% B; 12–37 min, 25–30% B; 37–40 min, 30–90% B; 40–45 min, 90% B; 45–50 min, 90–25% B; 50–65 min, 25% B).

### 4.6. Further Purification by Drowning-Out Crystallization and Preparative HPLC

Orientin, isoorientin, isovitexin, lucenin-1, lucenin-2, vicenin-1, and vicenin-2 were further purified by preparative HPLC with a specific program. For swertiajaponin, swertisin and vitexin, the 10 mg of swertiajaponin, 5 mg of swertisin, and 5 mg of vitexin were dissolved in 1 mL, 3 mL, and 4 mL of 90% methanol, respectively. Then, the solutions were transferred to a centrifuge tube. An equal volume of ultrapure water was added to the centrifuge tube, and the mixture was left overnight at 4 °C. Each sample underwent precipitation through drowning-out crystallization and was then subjected to centrifugation (10,000 rpm, 20 min) at 4 °C. After careful removal of the supernatant, the final product was obtained following washing with water and drying [30].

### 4.7. HPLC Analysis of Flavone C-Glycosides

The analytical method for determining preparative HPLC fractions and further purifying compounds involved the use of an analytical HPLC system (Dionex UltiMate 3000) with either a Luna^®^ Omega C18 column (250 mm × 4.6 mm × 5 µm) or a Kinetex^®^ Biphenyl column (250 mm × 4.6 mm × 5 µm). A gradient program was employed in the mobile phase, consisting of a combination of 0.2% (*v*/*v*) acetic acid in water (A) and methanol (B) as follows: 0–15 min, 5–20% A; 15–50 min, 20–40% A; 50–60 min, 40–5% A; 60–65 min, 5% A. The flow rate was set at 0.8 mL/min, and the injection volume was 20 μL. The column temperature was maintained at 30 °C, and signal detection was carried out at 280 nm using a diode array detector (DAD). The purity and the structural identification of compounds were performed with UV, MS, ^1^H NMR, and ^13^C NMR analysis and comparison with published data.

### 4.8. LC-MS Analysis of Flavone C-Glycosides

UPLC-MS analysis was conducted using an ACQUITY UPLC I-Class system coupled to a Vion IMS QTof (Ion Mobility Quadrupole Time-of-flight) hybrid mass spectrometer, which was equipped with an electrospray ionization (ESI) source (Waters, Manchester, UK). Then, 1 µL of each sample was injected, and the chromatographic separation was performed on a Kinetex Biphenyl column (Phenomenex, Torrance, CA, USA) (100 × 2.1 mm, 1.7 µm), maintained at 55 °C. The mobile phase flow was adjusted to 0.5 mL/min and a gradient elution transitioning from water with 0.1% formic acid (A) to methanol with 0.1% formic acid (B) was programmed as follows (A:B): 80:20 (t = 0 min), 80:20 (t = 0.5 min), 40:60 (t = 5 min), 10:90 (t = 6 min), 10:90 (t = 7 min), 80:20 (t = 7.5 min), 80:20 (t = 10 min). The ESI source was set to a 2.4 kV capillary voltage in negative ionization mode, with a 20 V sampling cone voltage. Source and desolvation temperatures were maintained at 120 °C and 450 °C, respectively. Nitrogen served as the desolvation and cone gas at flow rates of 800 and 50 L/h, respectively. For precise mass measurements, a lock mass correction was applied using the [M − H]—ion at *m*/*z* 554.2615 from a Leu-enkephalin solution (100 pg/µL in H_2_O/CH_3_CN (50/50 *v*/*v*) with 0.1% formic acid). The TOF operated in sensitivity mode, providing an average resolving power of 50,000 (FWHM). The MS spectra were recorded in profile mode across the 50–2000 *m*/*z* mass range with a scan time set to 0.2 s. For MS/MS experiments, argon served as collision gas, and the collision energy value was optimized to achieve a relative intensity between 10–20% for each selected precursor ion. Data acquisition was carried out using UNIFI software (V1.9.4, Waters).

### 4.9. Purity Determination and Structure Confirmation of Flavone C-Glycosides by NMR Analysis

Acquisition was carried out at 300 K on an Avance III 600 spectrometer (Bruker Biospin, Wissembourg, France). The ^13^C-NMR spectra were recorded at 150.91 MHz, and the ^1^H-NMR spectra were recorded at 600.17 MHz, using an inverse z-gradient triple-resonance (^1^H, ^13^C, ^15^N) probe head (TXI).

For quantitative NMR, 1 mg of each purified compound was transferred into 5 mm NMR tubes 509 (Norell isotope: NOR509UP7), with 800 µL of MeOH-D_6_. The sample was vortexed and sonicated for 10 min. Three replicates are realized. The 1D proton spectra with 90° flip was performed. Each spectrum consisted of 32 scans of 128 K data points with a spectral width of 8417 Hz (14 ppm) with a relaxation delay of 27.8 s (AQ and D1).

For identification of compounds, all replicates were pooled, dried, and solubilized with 800 µL of DMSO-D_6_ for each compound. The 1D proton spectra with 90° flip was performed. Each spectrum consisted of 64 scans of 128 K data points with a spectral width of 13,204 Hz (22 ppm). The 1D classical ^13^C and DEPTQ spectra were obtained using 16 K and 32 K scans, respectively, of 128 K data points with spectral widths of 37,878 Hz (250 ppm). The 2D COSY and TOCSY spectra were acquired with 2 scans per 1 K increments, and collected into 4 K data points, using spectral widths of 8417 Hz (14 ppm) in both dimensions. For the 2D HSQC and HSQC-TOCSY spectra, 64 scans per 8 K increments (the number of NUS sampling points was 512 complex points representing of 6.25% sampling density of 8 K points) were acquired, and collected into 2 K data points, with spectral widths of 8417 Hz (14 ppm) in F2 and 26,412 Hz (175 ppm) in F1. The 2D HMBC spectra were acquired with 128 scans per 8 K increments (the number of NUS sampling points was 512 complex points representing of 6.25% sampling density of 8 K points) and collected into 4 K data points, using spectral widths of 8417 Hz (14 ppm) in F2 and 37,732 Hz (250 ppm) in F1. Data treatment of all spectra was realized using Bruker Topspin software (version 3.6.2) and the calibration of the spectra was manually performed with the solvent residual signal [47].

### 4.10. In Vitro Antioxidant Assays

The antioxidant activity of flavone *C*-glycosides apigenin and luteolin was assessed using four different in vitro cell-free antioxidant assays: ABTS (2,2-azinobis 3-ethylbenzthiazoline-6-sulphonic acid), CUPRAC (cupric reducing antioxidant capacity), FRAP (Ferric Reducing Antioxidant Power), and iron chelating activity, as previously described by [48]. DMSO served as the blank and was the solvent used to solubilize the compounds, which were tested at concentrations ranging from 0.5 to 200 µM, while Trolox was used as the reference. The EC50 concentration represents the sample concentration required to scavenge 50% of free radicals. Each manipulation was repeated three times.

#### 4.10.1. ABTS Assay

The ABTS solution, composed of 7 mM ABTS and 2.45 mM potassium persulphate, was incubated in the dark for 16 h. Before the assay, the solution was diluted in ethanol and equilibrated at room temperature to achieve an absorbance of 0.7 at 734 nm in a microplate well. Subsequently, it was incubated for 15 min in the dark at 25 °C. For the assay, 10 μL of samples were incubated for 15 min with 190 μL of the ABTS^•+^ radical cation solution before measuring absorbance at 630 nm. The reaction between ABTS^•+^ and the compound is accompanied by the decolorization of the ABTS^•+^ as it is reduced back to its colorless neutral form.

The percentage of discoloration was calculated as follows:

% discoloration = [(A blank − A sample)/A blank] × 100(2)

where A sample is the absorbance of the sample and A blank corresponds to the absorbance of blank.

#### 4.10.2. Cupric Ion Reducing Antioxidant Capacity

The CUPRAC solution was composed of 10 mM Cu(II), 7.5 mM neocuproine, and 1 M acetate buffer (pH 7), mixed in a 1:1:1 (*v*/*v*/*v*) ratio. For the CUPRAC assay, 10 µL of samples was mixed with 190 µL of the CUPRAC solution in a microplate well and incubated for 15 min in the dark at 25 °C before measuring absorbance at 450 nm.

#### 4.10.3. Ferric-Reducing Antioxidant Power

The FRAP reagent was prepared freshly by mixing 10 mM 2,4,6-Tris(2-pyridyl)-1,3,5-triazine (TPTZ); 20 mM FeCl_3_·6H_2_O and 300 mM acetate buffer (pH 3.6), in a ratio 1:1:10 (*v*/*v*/*v*). For FRAP assay, 10 µL of samples was mixed with 190 µL of FRAP reagent in a microplate well, and incubated for 15 min in the dark at 25 °C before measuring absorbance at 630 nm.

#### 4.10.4. Iron Chelating Activity

The assay solution was prepared by mixing 20 mM ferric chloride (FeCl_3_) in acetate buffer (pH 4.9) and 6 mM ferrozine in water. Briefly, 10 µL of samples was mixed with 190 µL of chelation solution. The mixture was shaken vigorously and the absorbance was read 490 nm immediately after incubation for 30 min at room temperature.

### 4.11. Data Treatment

Data treatment was conducted to compare the results obtained in three replications, and the data were expressed as the mean ± SD. The half maximal effective concentration (EC50) value was chosen as a parameter for ABTS, and it was determined using the AAT Bioquest IC50 Calculator method. The Trolox equivalent antioxidant capacity (TEAC) value was chosen as a parameter for CUPRAC, FRAP, and ferrous-iron chelating assays. The non-parametric Wilcoxon tests were performed with the R software (v. 3.6.1, company Foundation for Statistical Computing, Vienna, Austria).

## 5. Conclusions

The present study employed a robust method to isolate and characterize 12 flavone *C*-glycosides from *Linum usitatissimum* L. aerial parts. The compounds isolated included orientin, isoorientin, vitexin, isovitexin, swertiajaponin, swertisine, vicenin-1, vicenin-2, lucenin-1, lucenin-2, carlinoside, and schaftoside. The isolation process involved an aqueous methanol extraction followed by purification using XAD-16N macroporous resin and preparative high-performance liquid chromatography (HPLC).

The antioxidant capacity was evaluated using multiple in vitro assays (ABTS, CUPRAC, FRAP, and iron chelation assay). These diverse assays provided a comprehensive assessment of the compounds’ antioxidant potential through different mechanisms.

Our results revealed that compounds derived from luteolin exhibited higher antioxidant activity compared to those derived from apigenin. This observation underscores the crucial role of the B ring in the flavonoid backbone for scavenging reactive oxygen species (ROS). The catechol structure (3′,4′-dihydroxy) in the B ring of luteolin derivatives indeed provides superior antioxidant capacity compared to the 4′-monohydroxy structure in apigenin derivatives. In contrast, glycosylation and methylation generally reduced the antioxidant potential of these compounds. The antioxidant properties were significantly influenced by several structural characteristics, the number and position of hydroxyl groups, planarity of the molecular skeleton, position and number of glycosyl groups and presence of methyl groups. These findings contribute to our understanding of the structure-function relationships in flavone *C*-glycosides and their potential as antioxidants.

In conclusion, the high content of *C*-glycosyl flavones in *Linum usitatissimum* L. and their significant antioxidant activity suggest potential applications in treating diseases associated with oxidative stress. However, further studies are necessary to elucidate the underlying mechanisms responsible for this antioxidant activity and to assess the role of flavone *C*-glycosides in contributing to it. Additionally, in vivo antioxidant assays are required to confirm the potential therapeutic applications of these compounds in disease treatment. These research directions could potentially lead to the development of new therapeutic approaches for oxidative stress-related diseases.

## Figures and Tables

**Table 1:** Purity (%) determination of flavone *C*-glycosides.

Compound	LC/MS Pos ^a^	LC/MS Neg ^a^	NMR ^b^
Vitexin	96.7 ± 0.6	97.4 ± 0.3	>99.5%
Isovitexin	94 ± 2.6	98.9 ± 0.4	>99.5%
Swertisin	99.3 ± 0.4	99.7 ± 0.1	>99.5%
Schaftoside ^c^	96.9 ± 0.9	98.2 ± 0.1	98.5 ± 0.2
Vicenin-1	93.3 ± 0.1	98.5 ± 0.3	99.2 ± 0.1
Vicenin-2	95.2 ± 1	97.9 ± 0.9	98.4 ± 0.1
Orientin	98.8 ± 0.8	99.5 ± 0.3	99.7 ± 0.03
Isoorientin	98.5 ± 0.8	99.0 ± 0.2	>99.5%
Swertiajaponin	99.3 ± 0.1	99.8 ± 0.2	>99.5%
Carlinoside	98.8 ± 1.3	99 ± 0.2	96.7 ± 0.1
Lucenin-1	98 ± 1.3	99 ± 0.3	95.7 ± 0.3
Lucenin-2	96 ± 1.9	99.1 ± 0.3	99.4 ± 0.2

The results are shown as the mean ± SD (*n* a + c = 2); (*n* b = 3).

## Data Availability

The original contributions presented in the study are included in the article/Supplementary Material, further inquiries can be directed to the corresponding authors.

## Supplementary Materials

The following supporting information can be downloaded at https://www.mdpi.com/article/10.3390/molecules29245829/s1, Supplementary Materials S1: Carbohydrate content in crude extract and XAD extract; Supplementary Materials S2: LC-MS information of flavone *C*-glycosides; Supplementary Materials S3: Structures and NMR tables of flavone *C*-glycosides; Supplementary Materials S4: Curves used to determine the EC50 for the ABTS test.
