# Supplementary material for: Relationship Between the Structure of the Flavone C-Glycosides of Linseed (Linum usitatissimum L.) and Their Antioxidant Activity
Source: Molecules. 2024 Dec 10;29(24):5829. doi: 10.3390/molecules29245829 (PMC11728773; doi:10.3390/molecules29245829)
Supplement: Supplementary file 1 [file molecules-29-05829-s001.zip › Supplementary Materials S4.pdf]

Supplementary Material S4: Curves used to determine the EC50 for the ABTS test (n=3). X represents the concentration in µM and Y the % of inhibition.

- **Orientin**

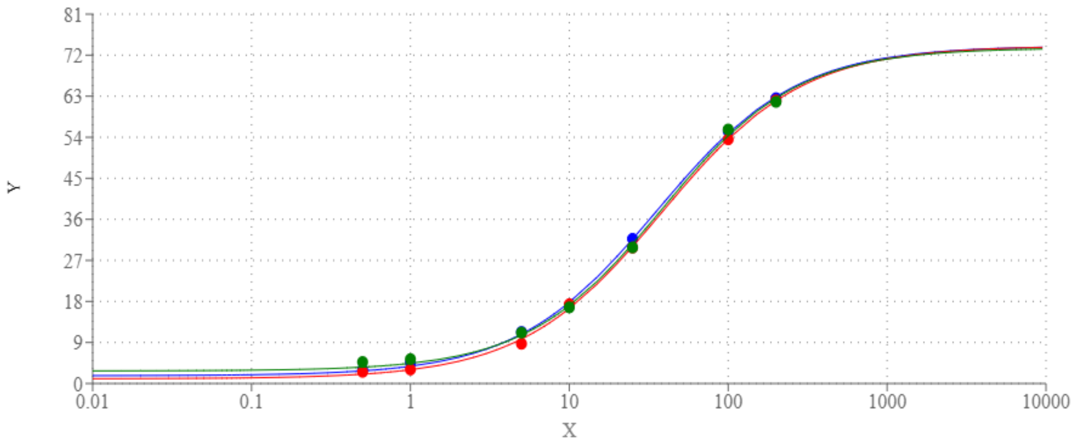

- **Isoorientin**

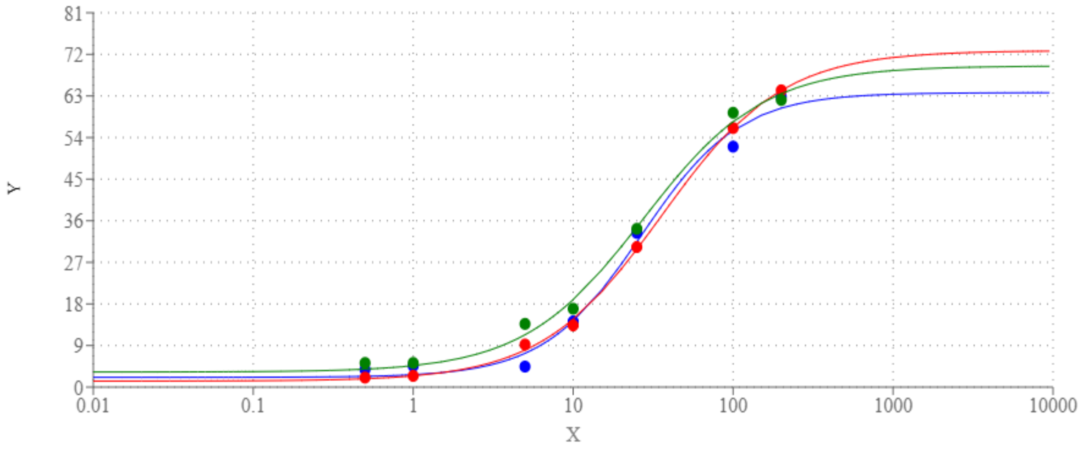

- **Swertiajaponin**

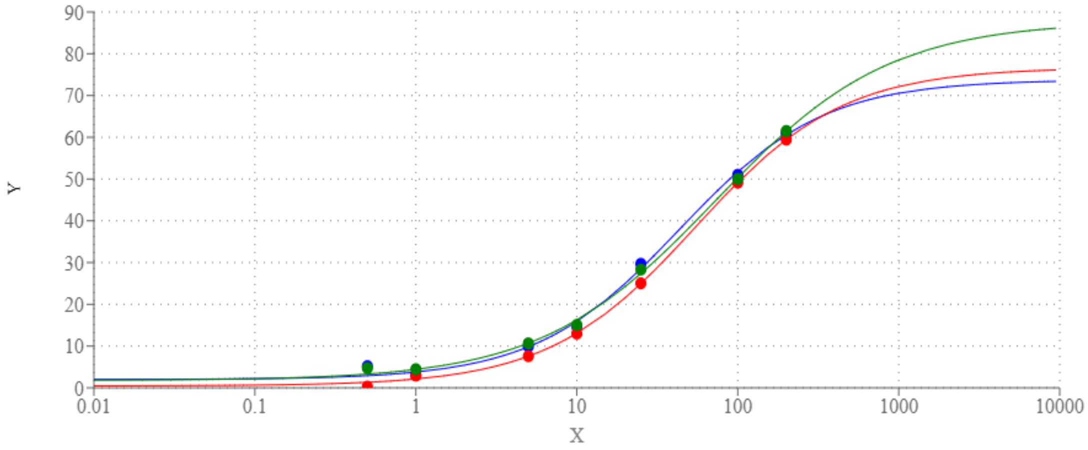

- **Lucenin-1**

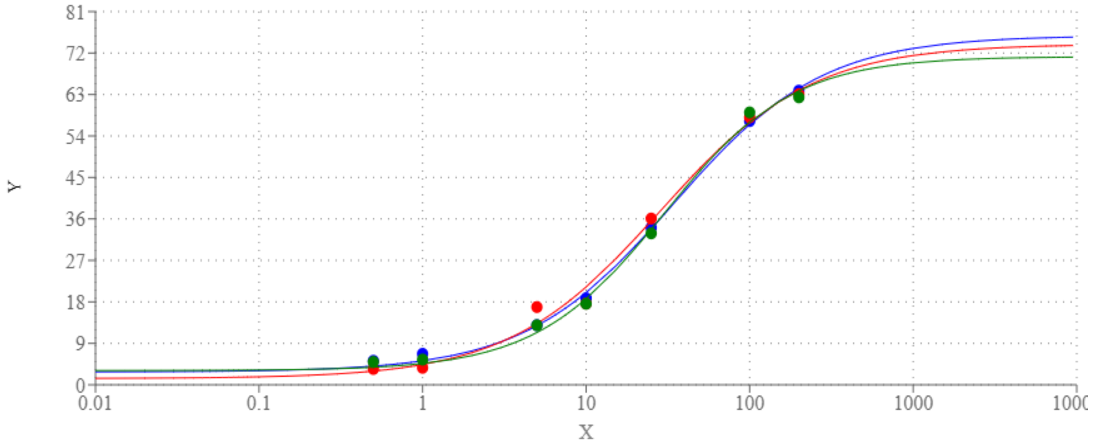

- **Carlinoside**

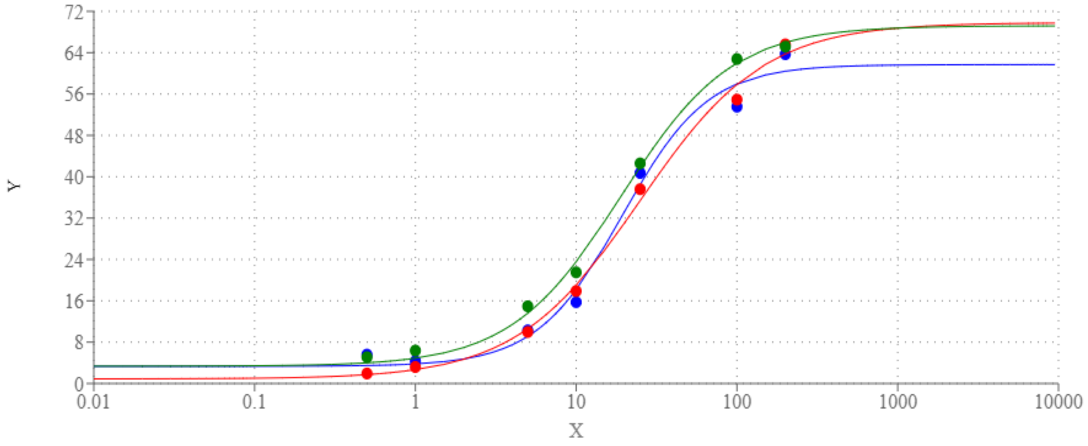

- **Lucenin-2**

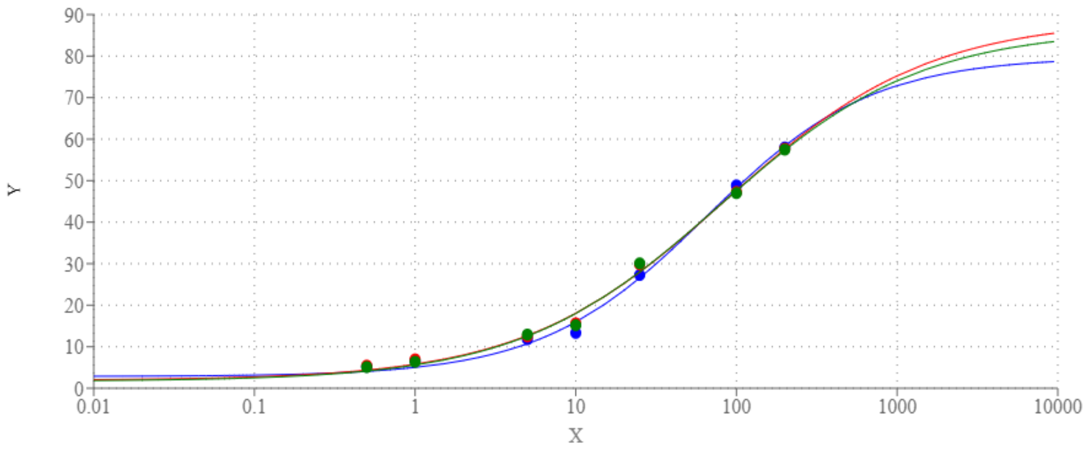

- **Luteolin**

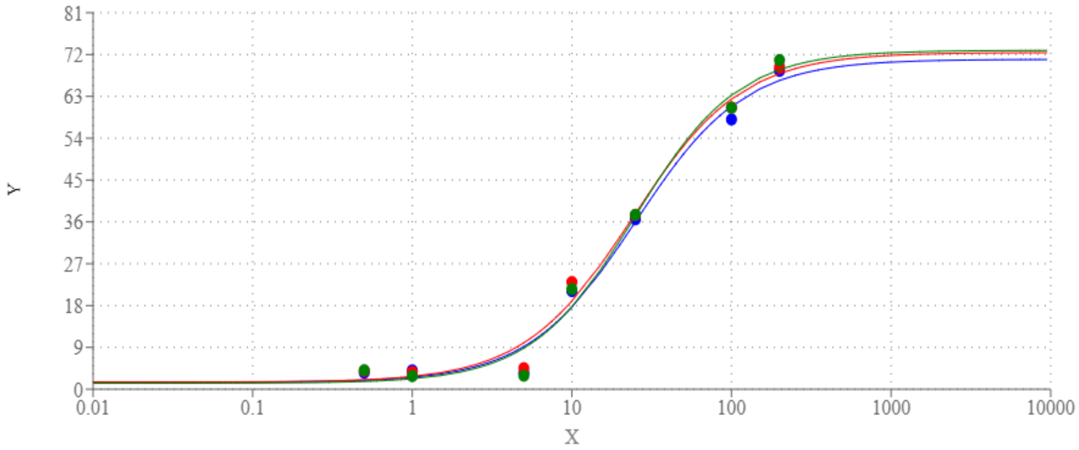

- **Trolox**

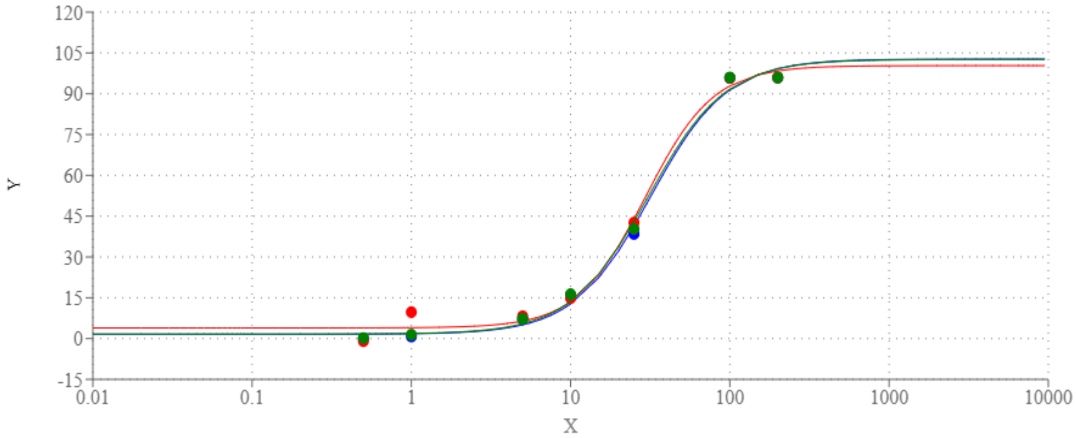

- **Vitexin**

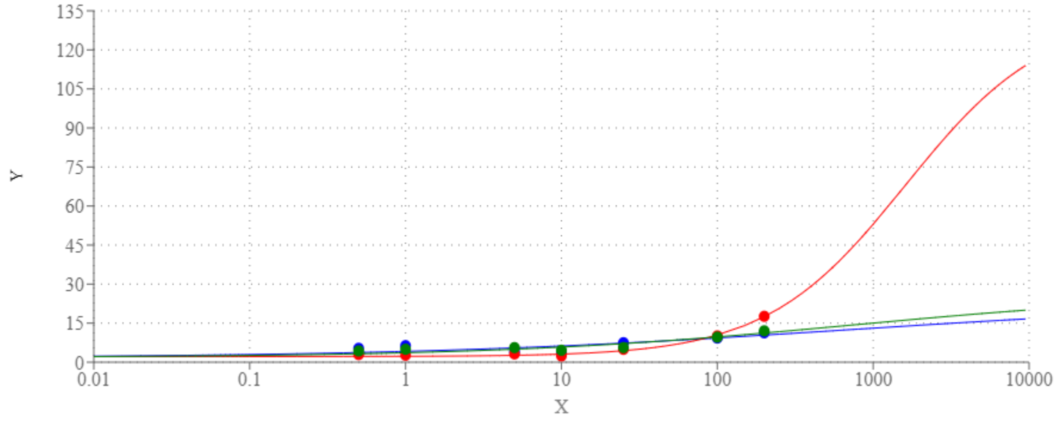

- **Isovitexin**

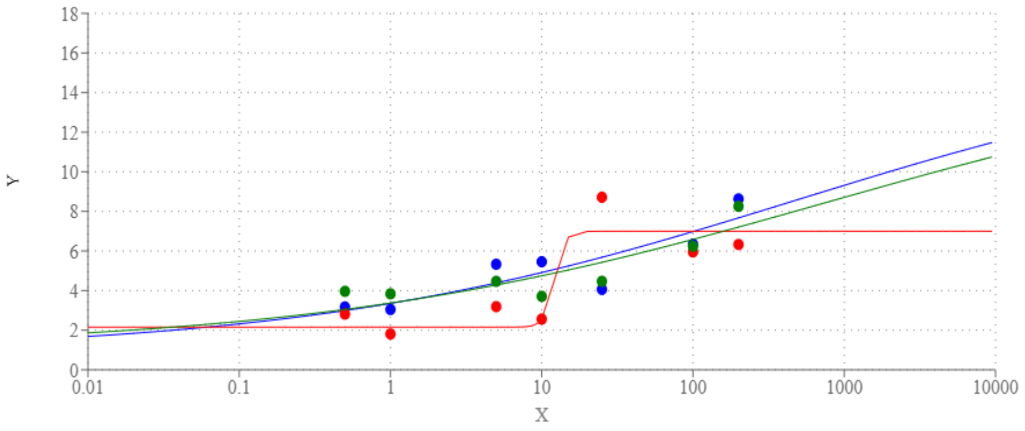

- **Swertisin**

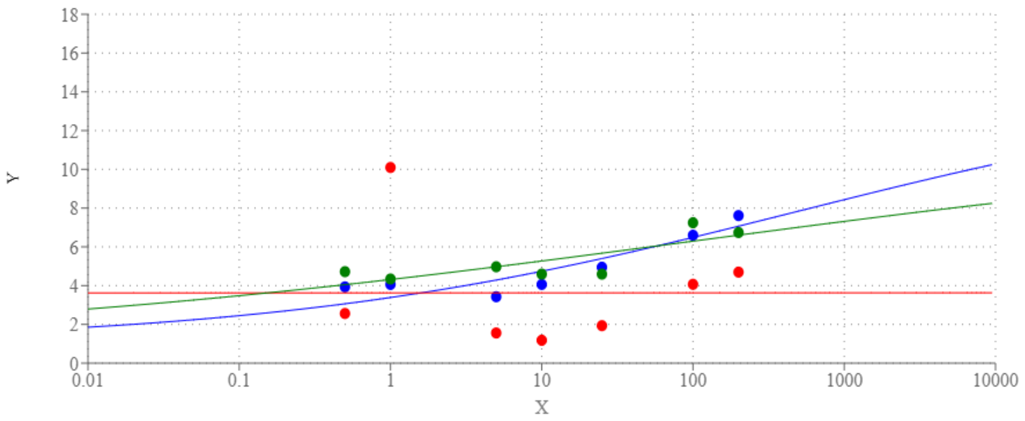

- **Vicenin-1**

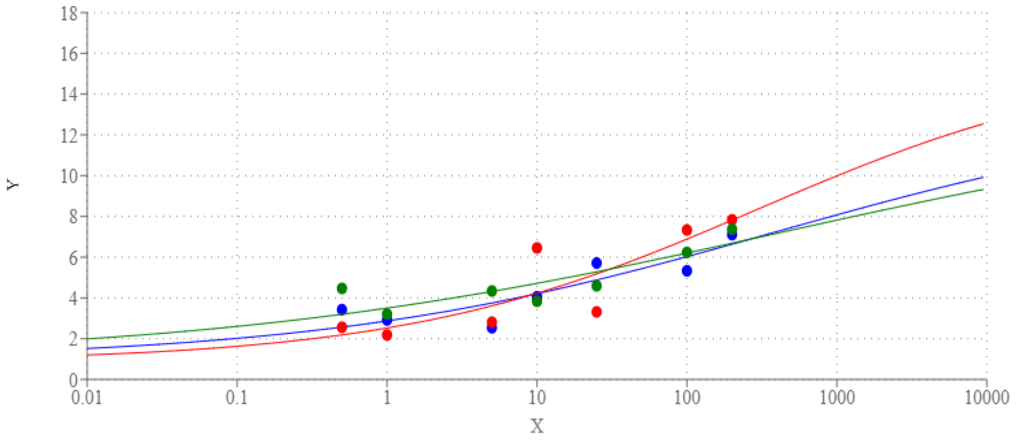

- **Schaftoside**

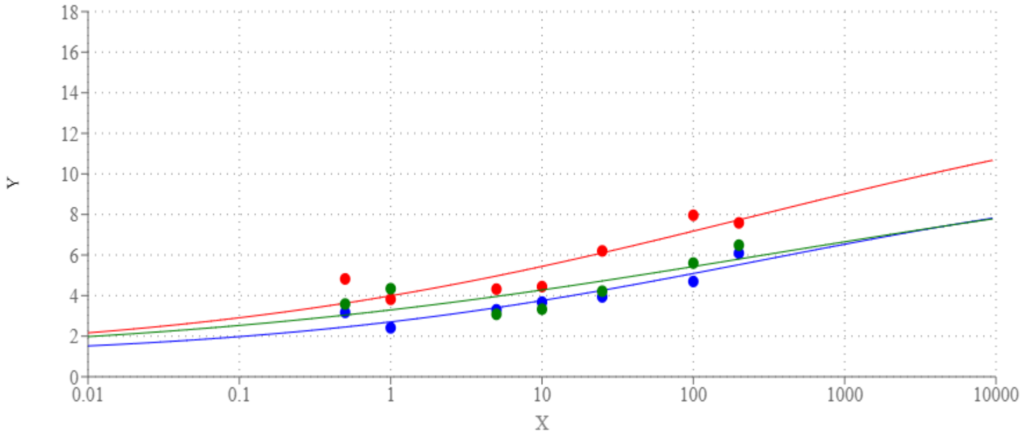

- **Vicenin-2**

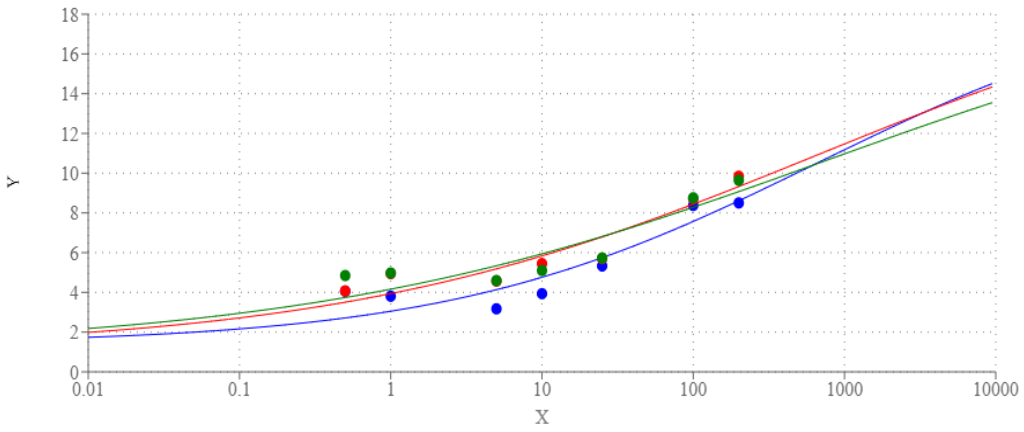

- **Apigenin**

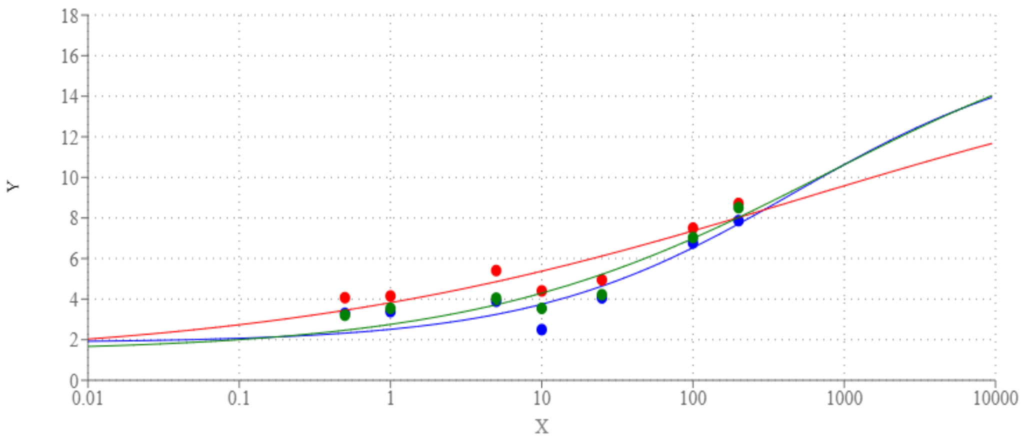

Y
X
0
2
4
6
8
10
12
14
16
18
0.01
0.1
1
10
100
1000
10000
