# Supplementary figures and images for: Relationship Between the Structure of the Flavone C-Glycosides of Linseed (Linum usitatissimum L.) and Their Antioxidant Activity
Source: Molecules. 2024 Dec 10;29(24):5829. doi: 10.3390/molecules29245829 (PMC11728773; doi:10.3390/molecules29245829)

**Supplementary Material S1:** Carbohydrates content in crude extract and XAD extract (n=3)

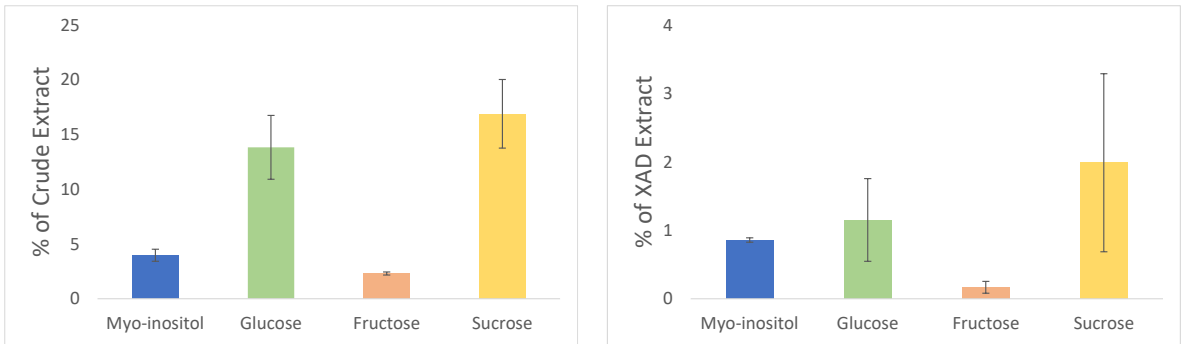

Supplement: Supplementary file 1 [file molecules-29-05829-s001.zip › Supplementary Materials S1.pdf]
